# Evaluating Carcinoembryonic Antigen and Glucose Levels in Pancreatic Cyst Fluid for Mucinous Versus Non-mucinous Differentiation

**DOI:** 10.7759/cureus.62686

**Published:** 2024-06-19

**Authors:** Seema R Sinha, Saptarshi Mondal, Md Jawed Akhtar, Rakesh Kumar Singh, Prem Prakash

**Affiliations:** 1 Biochemistry, Indira Gandhi Institute of Medical Sciences, Patna, IND; 2 General Surgery, Indira Gandhi Institute of Medical Sciences, Patna, IND; 3 Anatomy, Indira Gandhi Institute of Medical Sciences, Patna, IND; 4 Surgical Gastroenterology and Liver Transplant, Indira Gandhi Institute of Medical Sciences, Patna, IND

**Keywords:** non-mucinous cystic neoplasm, mucinous cystic neoplasm, pancreatic cystic neoplasm, glucose, carcinoembryonic antigen (cea)

## Abstract

Background: A more precise identification of mucinous cysts will lower the likelihood of needless pancreatic surgery. Pancreatic cyst fluid (PCF) contains glucose and carcinoembryonic antigen (CEA), which serve as biomarkers to differentiate mucinous from non-mucinous pancreatic cystic neoplasms (PCNs).

Objective: To evaluate the diagnostic accuracy of combined CEA and glucose levels in PCF for distinguishing mucinous from non-mucinous PCNs preoperatively.

Methods: After receiving approval from the Institutional Ethical Committee of Indira Gandhi Institute of Medical Sciences, Patna, a cross-sectional validation research was carried out. All patients ≥18 years of age who had undergone pancreatic surgery or endoscopic ultrasound-guided fine-needle aspiration (EUS-FNA) for a pancreatic cystic lesion and for whom PCF was acquired were eligible for inclusion. Patients were excluded if there was no PCF available, if they had been diagnosed with an extrapancreatic illness (such as ampullary adenoma), or if they could not be excluded due to pancreatic cancer generated from PCN. Diagnoses were pathologically confirmed. We performed measurements for CEA and glucose in PCF. CEA and glucose were measured using an Architect i2000SR analyzer (Abbott, Lake County, IL) and AU 5800 Beckman Coulter (Brea, CA), respectively. Diagnostic accuracy was evaluated by receiver operator characteristic (ROC) curves.

Results: PCF was obtained from 100 patients, of whom 54 (54%) had mucinous PCN and 46 (46%) had non-mucinous PCN. When CEA (cut-off ≥ 151 ng/ml) and glucose levels (cut-off ≤ 50 mg/dL) were combined, the results showed 46% sensitivity and 92% specificity. However, when CEA (cut-off ≥ 17 ng/ml) or glucose testing (cut-off ≤ 50 mg/dL) was used separately, the results showed 82% sensitivity and 73% specificity.

Conclusion: The combined CEA and glucose testing in PCF demonstrated high specificity and sensitivity for differentiating mucinous from non-mucinous PCNs, suggesting its potential utility in preoperative diagnosis.

## Introduction

Pancreatic cystic neoplasms (PCNs) are often reported as incidental findings during radiological imaging. In the general population, the age-related increase in the weighted incidence of PCN rises to 49% [1]. It affects a diverse range of lesions, including both benign and premalignant entities [2]. Current international standards state that mucinous PCNs should be followed up regularly or surgically resected as they are deemed pre-malignant [3,4]. Non-mucinous PCNs, however, do not require follow-up or surgical resection. Thus, it is imperative to accurately distinguish between mucinous and non-mucinous PCNs to prevent unnecessary surgery and the associated risks of death, morbidity, and costs. Differentiating between different varieties of PCNs is still difficult in day-to-day clinical practice. Only 72% of PCN cases are accurately identified, and only 86% of cases can adequately differentiate between mucinous and non-mucinous PCNs, even when best practices per clinical standards are followed [5]. Consequently, improving the ability to distinguish between mucinous and non-mucinous PCNs is essential for three things: (1) avoiding the needless lifelong follow-up of non-mucinous cysts; (2) facilitating early intervention in pre-malignant cases; and (3) avoiding unnecessary major abdominal surgery in cases where mucinous PCN is misdiagnosed. Pancreatic cyst fluid (PCF) obtained by endoscopic ultrasound-guided fine-needle aspiration (EUS-FNA) is routinely subjected to biochemical testing as part of diagnostic workups to improve diagnostic accuracy and differentiate between mucinous and non-mucinous PCNs [3]. Accessible biochemical markers are crucial for accurate PCF evaluation and everyday patient management from a clinical perspective. Carcinoembryonic antigen (CEA), which is frequently used for this purpose, can distinguish between mucinous and non-mucinous PCNs with a sensitivity of 52-73% and a specificity of 77-89% [6-8]. CEA has a cut-off value of 192 ng/mL. The ideal CEA cut-off value, however, is still up for discussion. Data from a recent individual patient meta-analysis involving 365 individuals [9] showed that the best diagnosis accuracy was obtained with a lower cut-off value of 20 ng/mL, with a sensitivity of 91% (95% CI: 88-94%) and specificity of 85% (95% CI: 72-93%). Glucose is a readily available biochemical PCN biomarker that is relatively new and shows promise. A recent meta-analysis reported a 94% diagnostic accuracy for distinguishing mucinous from non-mucinous PCNs [10], despite the variability in measurement methods (e.g., laboratory assays and glucometer testing) and the lack of standardization in clinical practice.

## Materials and methods

Study design and participants

To investigate the diagnostic accuracy of CEA and glucose in separating mucinous from non-mucinous PCNs, we carried out a cross-sectional validation study. The Standards for Reporting Diagnostic Accuracy Studies (STARD) were followed in the conduct of this study [11]. The Indira Gandhi Institute of Medical Sciences (IGIMS), Patna's Institutional Ethics Committee gave the study approval under reference number 1073/IEC/IGIMS/2023. Prior to the leftover material storage operation, all patients gave informed consent. The requirements for inclusion in this study included patients ≥ 18 years, patients in whom PCF was collected successfully, and patients who underwent pancreatic surgery or EUS-FNA for a pancreatic cystic lesion between July 2023 and April 2024. The patients were excluded if there was no PCF available, if they had been diagnosed with an extrapancreatic illness (such as ampullary adenoma), or if they had pancreatic cancer that was not developed from PCN.

Data collection

PCF samples were prospectively obtained at the pathology grossing department following pancreatic surgery or during EUS-FNA. Using standard techniques, endoscopic ultrasound (EUS) operations were carried out by a specialized gastroenterologist or under their direct supervision. Whether to aspirate cystic fluid directly from the lesion to obtain PCF was determined by the gastroenterologist. When the resection material was being processed at the pathology ward, PCF was taken straight out of the cystic lesion in patients who had undergone pancreatic surgery. Following receipt, the samples were refrigerated at -80°C by the pathology department. The sample taken during EUS was used for the analysis if more than one sample was taken from the same patient.

For CEA analysis, an aliquot of the PCF sample was transferred to the clinical lab following thawing at 37°C. All of the measurements were made in the clinical laboratory at IGIMS Patna. On the same day, measurements using chemiluminescence immunoassay were carried out on the Architect Abbott i2000SR (Abbott, Lake County, IL). Samples of glucose were rapidly thawed at 37°C. On the same day that samples were transferred to the clinical laboratory, measurements were taken using a Beckmann-Coulter AU 5800 autoanalyzer (Beckman Coulter, Brea, CA).

Mucinous PCN was composed of intraductal papillary mucinous neoplasm (IPMN) and mucinous cystadenoma (MCN). Based on histological analysis of the pancreatic surgical material, non-mucinous PCN was defined as pseudocysts, pancreatic neuroendocrine tumors, serous cystadenoma (SCN), ciliated foregut cysts, and lymphatic malformations.

Statistical analysis

The continuous data were presented as means and standard deviations, and the unpaired t-test was used to compare the data between the groups. Reports including categorical data were written using percentages or frequencies. Categorical data were compared using the chi-square test (or Fisher's exact test, if applicable). To compare areas under the curve (AUC), Fisher's exact test was employed. A p-value of less than 0.05 indicated that the result was statistically significant.

## Results

**Figure 1:**
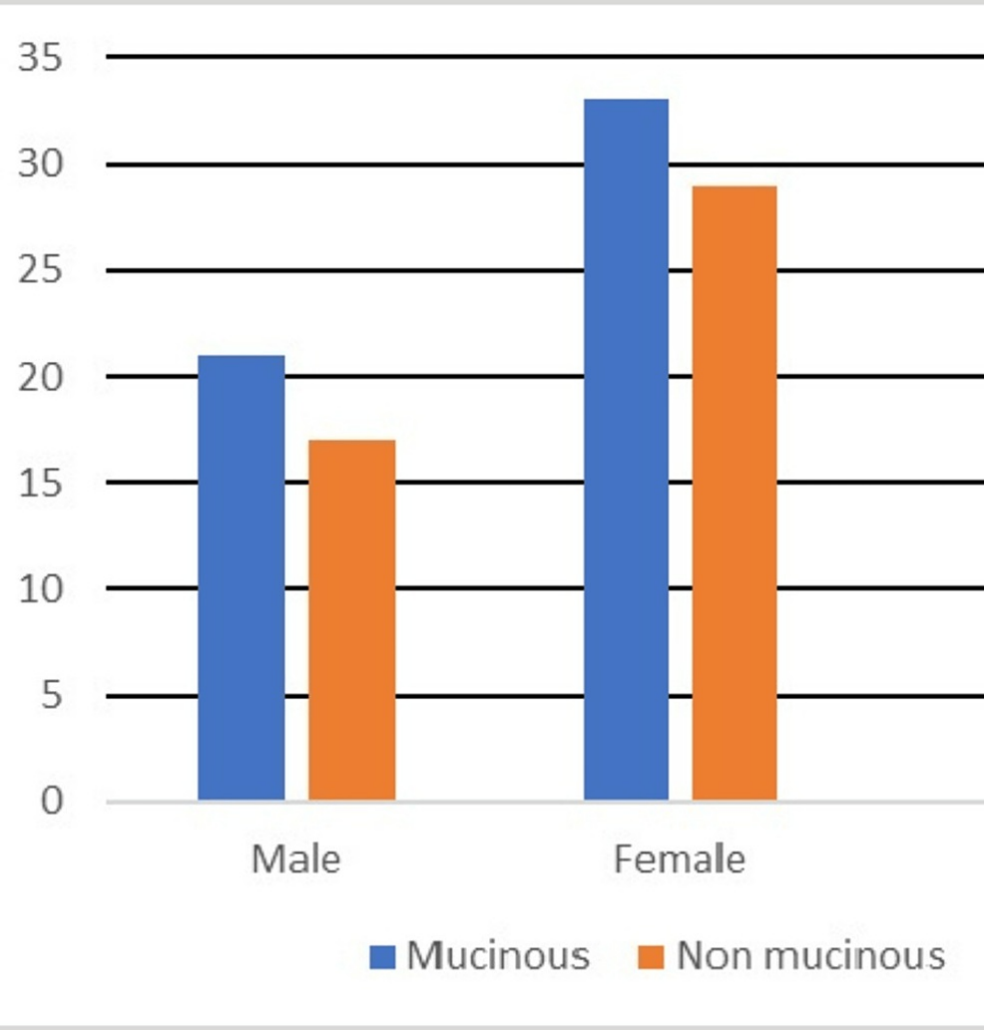
Bar chart showing the number of cases of mucinous and non-mucinous pancreatic cystic neoplasms among males and females.

A total of 112 patients were evaluated, of whom 100 were eligible and included in this study. Out of these 100 patients, 54 (21 males and 33 females) had mucinous PCNs, and 46 (17 male and 29 female) were diagnosed with non-mucinous PCNs (Figure 1).

Among pathologically confirmed mucinous PCN cases, 37 (68.51%) had IPMN and 17 (31.48%) had MCN; however, out of 46 non-mucinous PCNs, 20 (43.47%) had SCN, 18 (39.13%) had pseudocysts, three (6.52%) had pancreatic neuroendocrine tumors (pNET), two (4.34%) had solid papillary neoplasms (SPN), and three (6.52%) were diagnosed as having other benign type of disease. An overview of baseline and disease characteristics is displayed in Table 1.

**Table 1:** Baseline and disease characteristics. n: number; IQR: interquartile range; IPMN: intraductal papillary mucinous neoplasm; MCN: mucinous cystic neoplasm; SCN: serous cystadenoma; pNET: pancreatic neuroendocrine tumor; SPN: solid papillary neoplasm; CEA: carcinoembryonic antigen.

Parameters	Mucinous	Non-mucinous	P-value
Gender, n (%)			
Male	21 (38.88%)	17 (36.95%)	0.07
Female	33 (61.11%)	29 (63.04%)	
Age in years, median IQR	64.0 (55.0-73.0)	57.0 (42.0-68.0)	0.35
Cyst location			NA
Head	29 (53.70%)	25 (54.34%)	
Body	6 (11.11%)	9 (19.56%)	
Tail	19 (35.18%)	10 (21.73%)	
Multifocal	0	2 (4.34%)	
Final diagnosis, n (%)			
IPMN	37 (68.51%)	NA	
MCN	17 (31.48%)	NA	
SCN	NA	20 (43.47%)	
Pseudocyst	NA	18 (39.13%)	
pNET	NA	3 (6.52%)	
SPN	NA	2 (4.34%)	
Other benign	NA	3 (6.52%)	
Glucose (mg/dL), mean ± SD	19.557 ± 4.317	59.625 ± 5.241	0.0012
CEA (ng/mL), mean ± SD	120.03 ± 75.99	39.93 ± 56.65	0.0014

**Figure 2:**
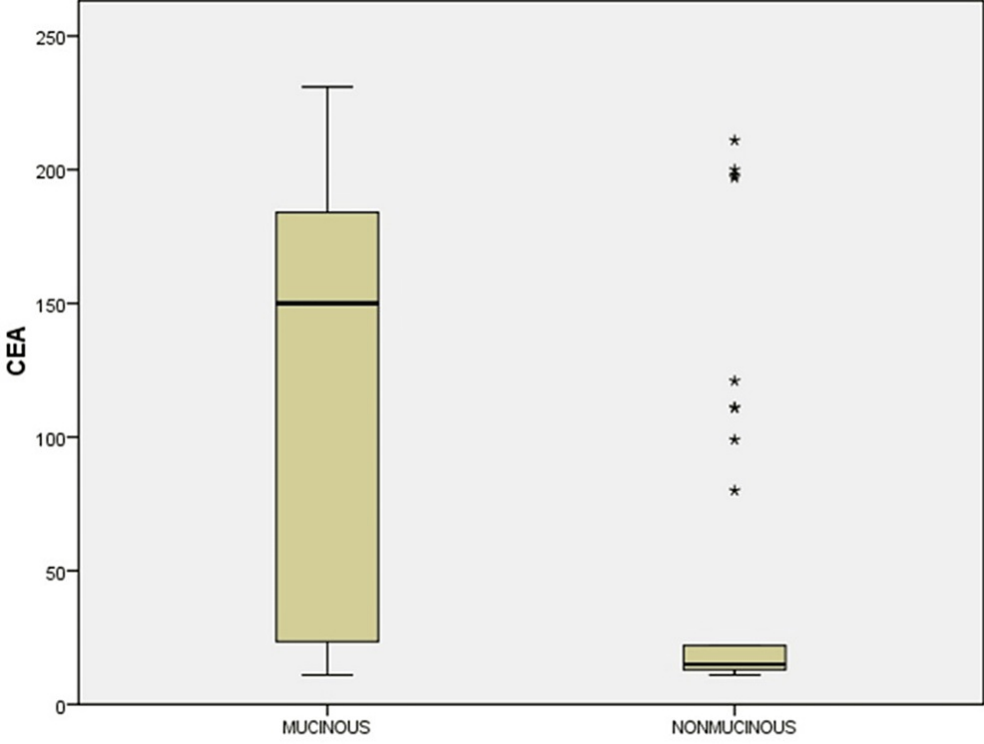
Box and Whisker plot showing CEA (ng/mL) levels in mucinous and non-mucinous PCNs. PCN: pancreatic cystic neoplasm; CEA: carcinoembryonic antigen; ng: nanogram; mL: milliliter.

Comparing mucinous PCN to non-mucinous PCN, the mean CEA was greater in the former (120 ± 75.9907 ng/mL; p = 0.0014) (Figure 2).

CEA and glucose cut‑off

**Figure 3:**
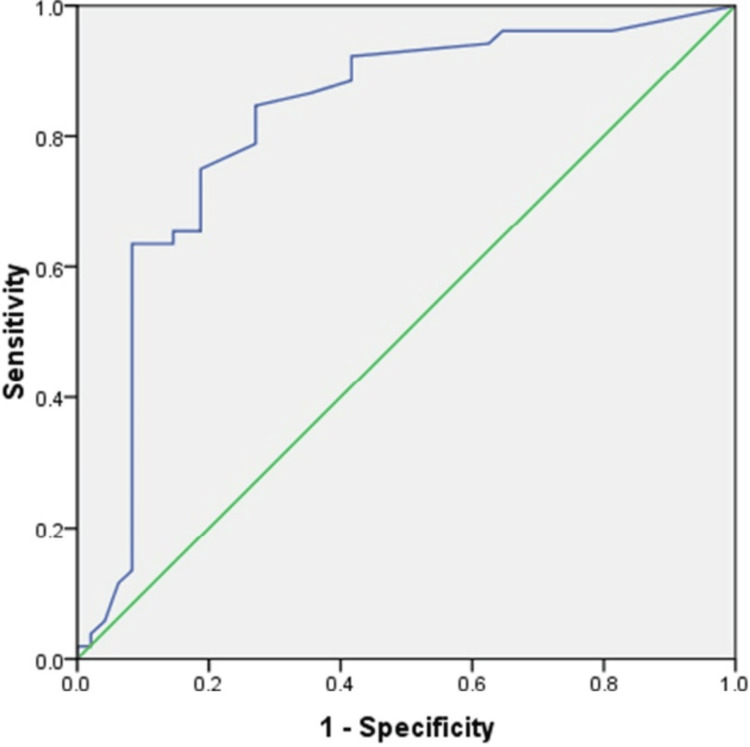
ROC curve analysis showing diagnostic performance of CEA in PCN. ROC: receiver operating characteristic; CEA: carcinoembryonic antigen; PCN: pancreatic cystic neoplasm.

A receiver operator characteristic (ROC) curve was drawn and showed that the AUC of CEA was 0.821 (95% CI: 0.733-0.91), indicating that mucinous and non-mucinous PCNs could be distinguished. There was a 54% sensitivity and 91% specificity at a cut-off value of ≥151 ng/mL. The sensitivity increased to 80% when the cut-off value was lowered to ≥17 ng/mL, but the specificity dropped to 58% (Figure 3).

**Figure 4:**
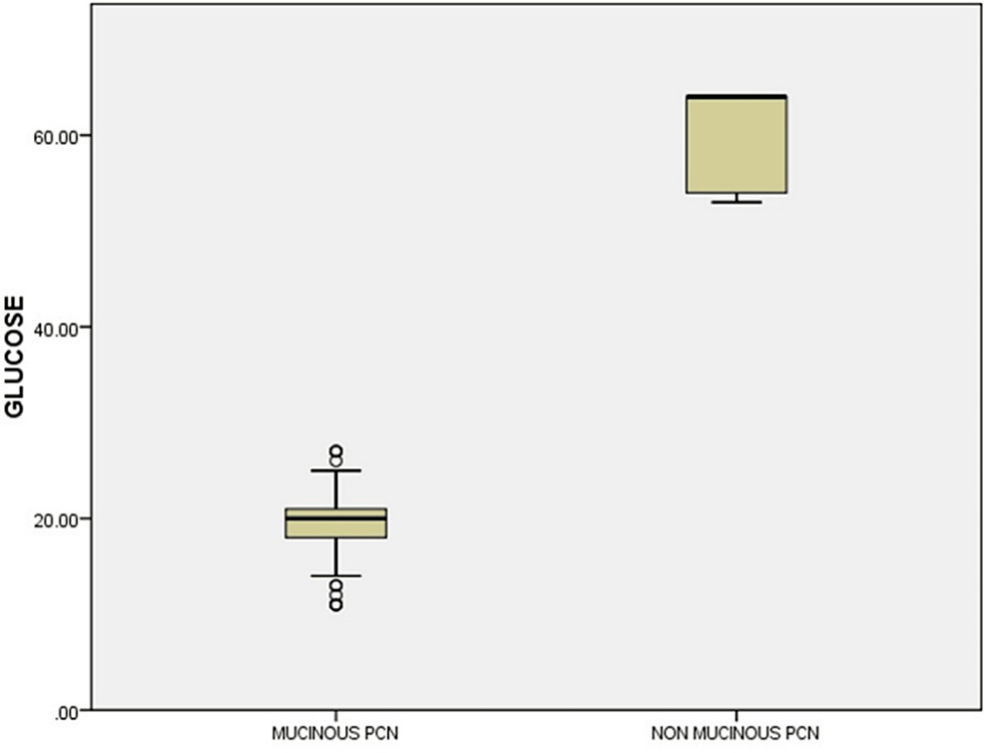
Box and Whisker plot showing glucose (mg/dL) level in mucinous and non-mucinous PCN. PCN: pancreatic cystic neoplasm; mg: milligram; dL: deciliter.

For mucinous cysts, the mean glucose level was 19.5576 ± 4.317 mg/dL, and for nonmucinous cysts, it was 59.625 ± 5.241 mg/dL (p = 0.0012) (Figure 4).

**Figure 5:**
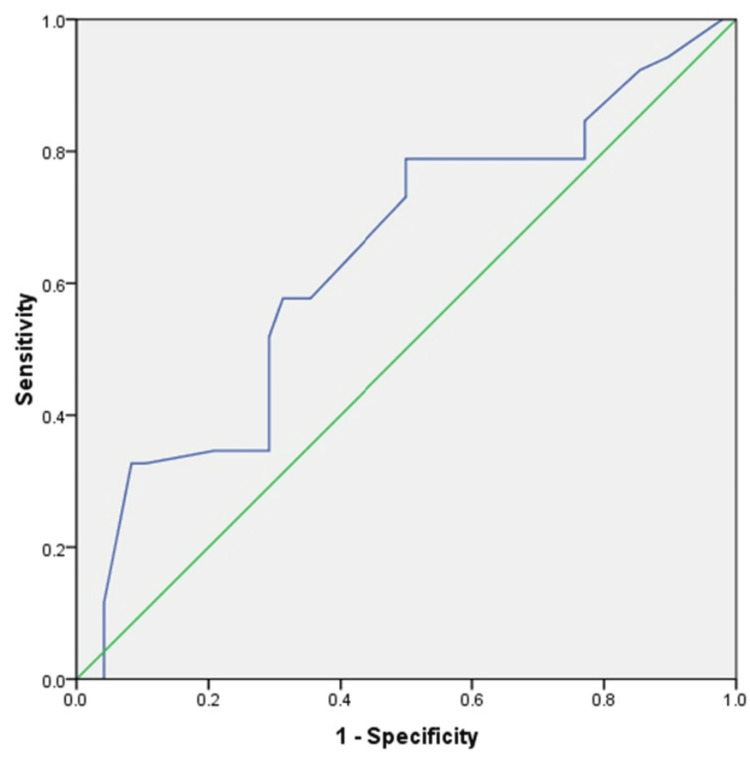
ROC analysis showing diagnostic performance of glucose in mucinous and non-mucinous PCNs. ROC: receiver operating characteristic curve; PCN: pancreatic cystic neoplasm.

ROC curve was drawn for glucose, which had an AUC of 0.648 (95% CI: 0.539-0.757) (Table 2). A specificity of 95% and a sensitivity of 50% was attained at a cut-off value of ≤ 50 mg/dL (Figure 5).

**Table 2:** CEA and glucose cut-off values to differentiate mucinous from non-mucinous PCNs. PCN: pancreatic cystic neoplasm; AUC: area under the curve; CI: confidence interval; CEA: carcinoembryonic antigen; dL: deciliter; mg: milligram; mL: milliliter; ng: nanogram; NPV: negative predictive value; PPV: positive predictive value.

Parameters	AUC	95% CI	Cut-off value	Sensitivity (%)	Specificity (%)	PPV (%)	NPV (%)
CEA	0.821	0.733-0.910	≥151 ng/mL	54	91	86	61
			≥17 ng/mL	80	58	71	73
Glucose	0.648	0.539-0.757	≤50 mg/dL	95	85	72	98
CEA and glucose	0.845	0.71-0.93	≥151 ng/mL and ≤50 mg/dL	46	92	84	60
			≥17 ng/mL and ≤50 mg/dL	82	73	75	72

The diagnostic accuracy of combined CEA and glucose testing in PCF to distinguish mucinous from non-mucinous PCNs was reported to have a sensitivity of 46% and a specificity of 92% for combined CEA and glucose testing ≥ 151 ng/mL and < 50 mg/dL, respectively. CEA and glucose ≥17 ng/mL and ≤50 mg/dL, respectively, have a sensitivity of 82% and specificity of 73%.

**Figure 6:**
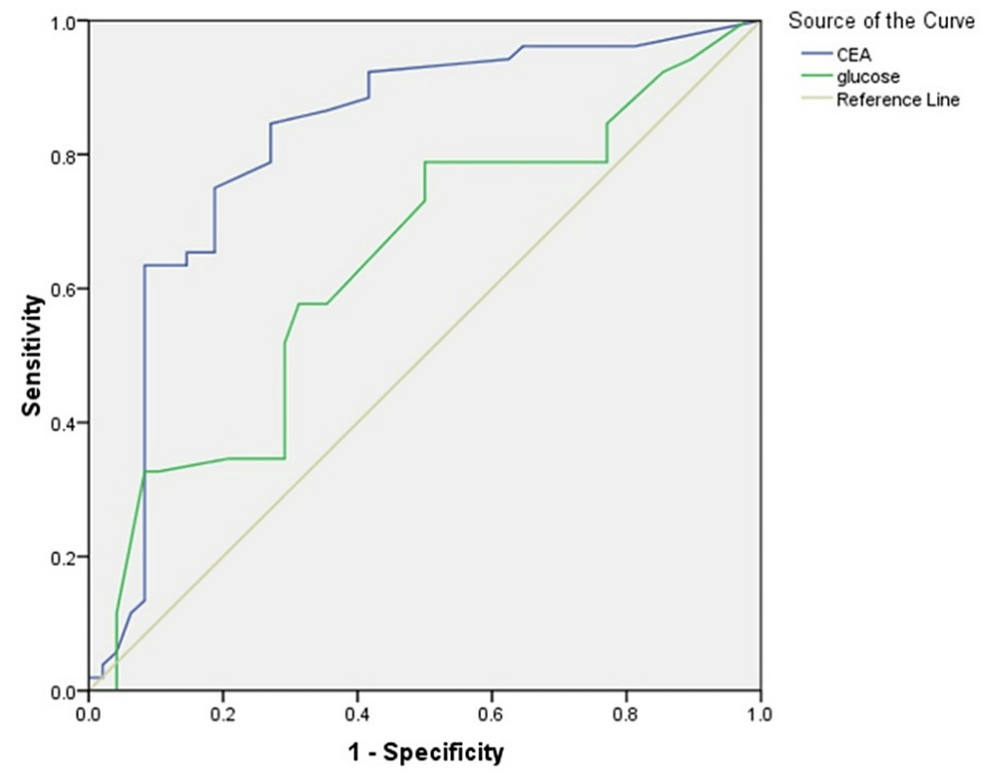
ROC analysis showing diagnostic performance of combined CEA and glucose in PCNs. ROC: receiver operating characteristic curve; PCN: pancreatic cystic neoplasm; CEA: carcinoembryonic antigen.

All of the biomarkers and cut-offs had a positive predictive value (PPV) of less than 90%, while the glucose measurement showed a negative predictive value (NPV) of 98% (high glucose excludes a mucinous cyst). Excellent specificity and sensitivity were achieved when combining CEA and glucose tests in PCF to differentiate between mucinous and non-mucinous PCNs. When used in conjunction with the updated CEA cut-off (≥17 ng/mL), glucose testing demonstrated >95% sensitivity and 82% NPV for mucinous cysts. In contrast, solely glucose reached an NPV of 98% (Figure 6).

## Discussion

The diagnostic accuracy for differentiating mucinous from non-mucinous PCNs using both CEA and glucose testing of PCF was investigated in this validation study. The results of the study showed that a high degree of specificity and sensitivity was attained when CEA and glucose testing were combined. The implementation of the revised threshold for CEA (≥17 ng/mL) resulted in an improved sensitivity of 80%. The glucose ≤50 mg/dl testing demonstrated a sensitivity of 95% and an NPV of 98%, indicating that it is a highly reliable biomarker that can be readily incorporated into clinical practice.

The AUC for combined CEA and glucose measurement was 0.94 (95% CI: 0.88-0.99), with 88% sensitivity and 93% specificity, according to a retrospective research that included 102 patients [12]. According to two systematic reviews, glucose in PCF can effectively distinguish between mucinous and non-mucinous PCNs [10,13]. When compared to glucose alone, McCarty et al.'s examination of the combination of CEA and glucose showed no improvement in diagnostic value. However, only four research articles were included in this systematic review analysis [10]. In contrast, the two systematic reviews demonstrated good specificity (86.6% and 88%) and sensitivity (91% and 90.5%) for glucose testing. Our findings also demonstrated a high 95% glucose sensitivity and a comparable 83-66% specificity. When compared to other investigations, the results of this investigation support other studies that looked at the diagnostic accuracy of CEA using the traditional threshold of ≥192 ng/mL. PCN that is mucinous and non-mucinous are distinguished using threshold values. The sensitivity rates reported in these studies varied from 52% to 73%, while the specificity rates ranged from 77% to 89% [6-8]. However, the ideal threshold value for CEA is still a topic of discussion, as indicated by a recently conducted meta-analysis of individual patient data.

The capacity to do next-generation sequencing for molecular analysis is a recent development in PCF analysis. According to a recent investigation, the presence of mutations in GNAS and KRAS provides a 97% diagnostic accuracy for mucinous PCN [14]. Even though the results seem encouraging, it is crucial to remember that mutation sequencing is a relatively new technology and that using it requires skilled laboratory personnel. Thus, the ability to access biochemical indications quickly remains crucial for the delivery of clinical patient care. Myrte Gorris and colleagues conducted a study. Collectively, CEA and glucose tests in PCF demonstrated exceptional specificity and sensitivity in differentiating between mucinous and non-mucinous PCNs. The only substance with an NPV > 95% was glucose, yet glucose testing produced >95% sensitivity for mucinous cysts when used either by itself or in combination with the updated CEA cut-off (≥20 ng/mL) [15].

There are certain limitations in this study. Initially, it should be noted that the sample size was comparatively limited. Nevertheless, this group accurately represents the patient population typically encountered in clinical practice. Additionally, the fact that the PCF samples were only obtained in one center for tertiary care may restrict the findings' applicability in other hospital settings. Moreover, PCF was obtained during both surgical and EUS-FNA procedures, which might have caused differences in the samples. This is because surgical samples were moved to the grossing room and then kept at -80°C after PCF was collected. It is unlikely that there were any notable changes in glucose and CEA levels at this time, though, given the brief transit duration.

However, the notable advantages of this study include the utilization of a cohort of PCF samples that were obtained prospectively, and the study population accurately reflects everyday clinical practice. Furthermore, endoscopic PCF samples were promptly frozen at a temperature of -80°C upon collection, thereby minimizing the possibility of degradation. This study offers valuable information on the diagnostic precision of various testing methods, allowing clinicians to consider the most effective combination. The primary benefit of combining CEA and glucose testing is the ability to utilize specific combinations that yield the most convenient results for each individual patient. For instance, in a patient with multiple concurrent medical conditions and a need for surgical removal, it is crucial to have a high level of specificity to confirm the presence of a mucinous cyst. In this situation, it may be recommended to conduct combined testing using glucose (≤50 mg/dL) and CEA (≥151 ng/mL). On the other hand, a high degree of sensitivity is necessary to firmly rule out the existence of a mucinous cyst in patients who might no longer need follow-up. A testing strategy that combines either a positive CEA (≥17 ng/mL) or a positive glucose (≤50 mg/dL) can be utilized. However, it is important to undertake bigger cohort studies to create a design.

## Conclusions

Overall, there was a significant degree of sensitivity and specificity shown by the combination of CEA and glucose tests in PCF, suggesting that it might be worked into routine clinical practice. An elevated threshold of CEA improved the accuracy of the diagnosis. Glucose testing has demonstrated a high level of sensitivity, so it may be used for differentiating between non-mucinous and mucinous PCN prior to surgery.
